# Association between Platelet to Neutrophil Ratio (PNR) and Clinical Outcomes in STEMI Patients after Successful pPCI: A Secondary Analysis Based on a Cohort Study

**DOI:** 10.1155/2022/2022657

**Published:** 2022-02-24

**Authors:** Hao Wang, Xiaochun Qing, Hua Wang, Yunfei Gu

**Affiliations:** ^1^Department of Cardiology, Luoyang Central Hospital Affiliated to Zhengzhou University, Luoyang, Henan Province, China; ^2^Ultrasound Department, Luoyang Central Hospital Affiliated to Zhengzhou University, Luoyang, Henan Province, China

## Abstract

**Purpose:**

This study was aimed at investigating whether the platelet-to-neutrophil ratio (PNR) is independently related to the prognosis of patients with ST-elevation myocardial infarction (STEMI) after successful primary percutaneous coronary intervention (pPCI).

**Methods:**

This was a secondary analysis of data retrieved from the DATADRYAD database, which was a prospective cohort study. A total of 464 STEMI patients who underwent successful pPCI were recruited between January 2010 and October 2014. The target-independent variable, PNR, was measured at the baseline. The dependent variable in the current study was the occurrence of major adverse cardiovascular events (MACEs) during the 30-month follow-up.

**Results:**

Two patients were excluded from the final analysis because their platelet counts were unavailable. The average age of the 462 participants was 63 ± 11.92 years, and approximately 76.6% were male. After adjusting for age, sex, anterior wall myocardial infarction (MI), history of MI, apelin-12, apelin-12 change rate, left ventricular end-diastolic diameter, peak cardiac troponin I, pathological Q wave, Killip classification grade, fasting blood glucose, albumin, GENSINI score, and estimated glomerular filtration rate, a nonlinear relationship was found between the PNR and MACEs in the included cohort. The threshold value of the PNR for MACEs was 23.1. Over this cutoff value, the incidence rate of MACEs increased by 43% per 10-unit change in PNR (95% CI: 1.16–1.75, *p* = 0.0006).

**Conclusion:**

There was a threshold relationship between PNR and MACEs in patients with STEMI who underwent successful pPCI. The incidence of MACEs was positively associated with the PNR when the PNR exceeded 23.1.

## 1. Introduction

ST-segment elevation myocardial infarction (STEMI) is the most severe type of heart attack [1]. It remains the most common cause of death in adults globally [2]. Although the rates of immediate STEMI-related deaths have declined because of technological advances and the widespread use of reperfusion therapy, the risk of mortality associated with STEMI remains high, especially for high-risk patients [3]. As indicated by the latest guidelines by the European Society of Cardiology (ESC) for the management of STEMI, the in-hospital mortality and one-year mortality rates for STEMI are approximately 10% [4]. Post-STEMI survival is influenced by clinical factors, such as age, sex, infarct area, and biochemical factors [5]. Therefore, earlier identification of high-risk patients makes it possible to provide more aggressive treatment and closely monitor to those patients, especially after discharge. There is no doubt that this is of great significance.

Complete blood count (CBC) is the first test on admission for all patients. It is easy to perform, inexpensive, and rapid. Recently, the absolute blood cell counts or the ratios between them have received increased clinical attention as inflammatory markers [6–9]. The predictive value of the platelet-to-lymphocyte/neutrophil-to-lymphocyte ratio (PLR/NLR) and the systemic immune-inflammation index (platelet count × NLR) were demonstrated to affect the clinical outcomes of STEMI patients [8–13]. However, few studies have focused on the association between the platelet-to-neutrophil ratio (PNR) and the prognosis of STEMI patients [8]. One research letter by Somaschini et al. suggested that the PNR may be associated with the prognosis of STEMI patients undergoing primary percutaneous coronary intervention (pPCI) [14]. Platelets are inducers and biomarkers of chronic inflammatory conditions such as atherosclerosis and atherothrombosis [15]. Neutrophils are acute inflammatory markers, as they are the first cells to be recruited to the myocardium under the condition of myocardial infarction [16]. To a certain extent, the PNR reflects the balance between chronic and acute inflammatory condition [14]. Therefore, this study aimed to investigate whether PNR is independently associated with the occurrence of major adverse cardiovascular events (MACEs) in patients with STEMI who underwent successful pPCI.

## 2. Participants and Methods

### 2.1. Data Source

The data used in the current study were obtained from an open-access database, DATADRYAD website (https://datadryad.org/), which allowed secondary analysis of their raw data with suitable citation [17]. The database file included the following variables for further analysis: age, sex, hypertension, diabetes mellitus (DM), previous myocardial infarction (MI), anterior wall MI, systolic blood pressure (SBP), heart rate (HR), white blood cell (WBC) count, neutrophil percentage, hemoglobin, platelet count, blood urea nitrogen (BUN), creatinine, uric acid, albumin, fasting blood glucose (FBG), triglyceride (TG), total cholesterol (TC), high-density lipoprotein cholesterol (HDL-C), low-density lipoprotein cholesterol (LDL-C), peak cardiac troponin I (cTnI), peak CK-MB levels, D-dimer levels, left ventricular end-diastolic diameter (LVEDD), apelin-12, apelin-12 change rate, culprit vessels, stent number, pathological Q wave, Killip classification grade, and GENSINI score. We calculated the PNR for every individual using the equation:

(1) $$\begin{array}{c} \text{PNR}=\frac{\text{platelet count}}{\text{WBC}\times \text{percentage of neutrophils}} \end{array}$$

The estimated glomerular filtration rate (eGFR) was estimated using the Modification of Diet in Renal Disease (MDRD) equation adjusted for Chinese populations [18]:

(2) $$\begin{array}{c} \text{eGFR }\left(\text{mL}/\text{s per }1.73\;{\text{m}}^{2}\right)=175\times {\left(\text{serum creatinine in mmol}/\text{L}\right)}^{-1.234}\times {\left(\text{age in years}\right)}^{-0.179}\times \left(0.79\text{ if the patient wasis female}\right) \end{array}$$

### 2.2. Study Design and Population

The original study reported by Yang et al. was designed as a prospective cohort study to address the relationship between serum apelin-12 and MACEs (cardiac death, reinfarction of current culprit vessels, clinically driven target lesion revascularization (TLR), cardiogenic shock, and congestive heart failure) in STEMI patients after successful pPCI [17]. They consecutively enrolled STEMI patients who met the diagnostic criteria for STEMI and were admitted to the First People's Hospital of Taizhou, Zhejiang, China, between January 2010 and October 2014. The diagnostic criteria included persistent chest pain beyond 30 minutes, ECG alterations, and elevated myocardial enzyme and troponin levels. All enrolled patients underwent successful standard pPCI as described by Yang et al. [17]. Written informed consent was obtained from each participant prior to data collection. After applying the exclusion criteria, 108 patients were excluded, as described by Yang et al. [17]. In the current study, we used PNR as the target-independent variable and found some predictive value for MACEs in a 30-month follow-up period. The covariates considered in this study comprised demographic data and potential variables that could affect PNR or MACEs based on previous studies and our clinical experience. The following variables were used in the fully adjusted model: age, sex, anterior wall MI, history of MI, apelin-12, apelin-12 change rate, LVEDD, peak cTnI level, pathological Q wave, Killip classification grade, FBG and albumin levels, GENSINI score, and eGFR screened by univariate analysis.

### 2.3. Statistical Analysis

Continuous variables are presented as mean ± standard deviation for normally distributed data or median (interquartile range [IQR]) for nonnormally distributed data. The Kolmogorov-Smirnov test was used to determine whether the data were normally distributed. Categorical variables were expressed as frequencies and percentages. Data were grouped based on quartiles of PNR values, and the differences among the groups were detected using *χ*^2^ (categorical variables), one-way ANOVA (for normally distributed data), or the Kruskal-Wallis H test for skewed distributed variables. The data analysis was based on the following principles: (1) whether any relationship exists between the PNR and MACEs and determining the nature of that relationship (linear or nonlinear), (2) estimating the factors affecting the association between PNR and the prognosis of STEMI patients, and (3) determining the true relationship between PNR and MACEs after adjustment for potential interference factors. The data analysis can be summarized in two steps. *Step 1*: Constructing three univariate and multivariate Cox proportional hazard models as follows: model 1, the crude model, in which no covariate was adjusted; model 2, the minimally adjusted model, in which only the age and sex were adjusted; and model 3, the fully adjusted model, in which all potential covariates screened out were added to model 2. During data analysis, the PNR was converted into a categorical variable (PNR quartiles) to confirm the stability of the results. To observe the existence of a nonlinear relationship, the trend of the *p* values was obtained by calculating the regression coefficient of the *p* values after substituting the median PNR values of the four groups using the Cox regression equation as a continuous variable. *Step 2*: If the nonlinearity was captured, a Cox proportional hazard regression model with cubic spline functions and smooth curve fitting (penalized spline method) was created. First, we calculated the inflection point using a recursive algorithm and then constructed a two-piecewise Cox proportional hazard model on both sides of the inflection point. Subsequently, the log-likelihood ratio test was used to verify the most suitable model for the association between PNR and MACEs. All analyses were performed using the statistical software R (https://www.R-project.org, The R Foundation) and EmpowerStats (http://www.empowerstats.com, X&Y Solutions, Inc., Boston, MA, USA). Statistical significance was set at *p* < 0.05 (two-sided).

## 3. Results

### 3.1. Baseline Characteristics of the Selected Participants

After excluding two patients (ID 42 and 46) as their platelet counts were missing, the data of 462 patients (mean age, 63 ± 11.92 years; 76.6% were male, *n* = 354) were finally analyzed. The baseline characteristics of the participants are shown in Table 1, according to the PNR quartile. No significant differences were detected in most of the analyzed variables among the different PNR groups, except in the WBC count, neutrophil percentage, platelet count, LDL-C, albumin levels, and survival time. The participants with the highest PNR (43.41–78.74) had the shortest survival duration (*p* = 0.006).

### 3.2. Univariate Analysis

In univariate analysis, male sex (HR = 1.55, 95% CI: 1.03–2.33), PNR (HR = 1.02, 95% CI: 1.01–1.04), stent number > 3 (HR = 4.63, 95% CI: 1.64–13.10), creatinine (HR = 1.02, 95% CI: 1.01–1.03), and uric acid (HR = 1.00, 95% CI: 1.00–1.00) were associated with an increase in MACEs (Table 2). The WBC level (HR = 0.84, 95% CI: 0.79–0.89) was negatively associated with MACEs.

### 3.3. Unadjusted and Adjusted Cox Proportional Hazard Model

Three models were constructed as described previously to detect the independent effects of PNR on MACEs. The effect size of PNR was very stable among the different models, as shown in Table 3. For the sensitivity analysis, PNR was converted into a categorical variable (PNR quartiles), and the trend of *p* values in different models was consistent with the result achieved with the continuous PNR values. Moreover, the trend of the effect size in the different PNR groups was nonequidistant, which indicated the possibility of nonlinearity.

### 3.4. Nonlinearity of PNR and MACEs

**Figure 1:**
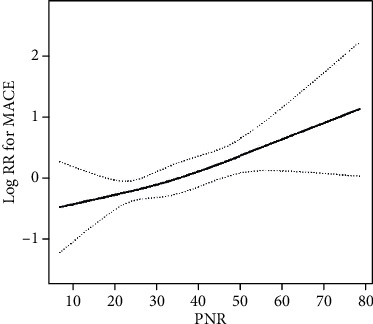
The impact of PNR on MACEs of STEMI patients after pPCI. A nonlinear relationship between them was detected after adjusting for age, sex, anterior wall myocardial infarction, myocardial infarction history, apelin-12, apelin-12 change rate, LVEDD, peak cTnI, pathological Q wave, Killip grade, FBG, albumin, GENSINI, and eGFR.

A nonlinear relationship between PNR and MACEs was found in the current study after adjusting for age, sex, anterior wall MI, MI history, apelin-12, apelin-12 change rate, LVEDD, peak cTnI, pathological Q wave, Killip classification grade, FBG, and albumin levels, GENSINI score, and eGFR (Figure 1). The log likelihood ratio test *p* value was <0.05; therefore, a two-piecewise Cox proportional hazard model should be more accurate in representing the association between PNR and MACEs. The inflection point was at 23.1. Given that there is no clinical reference range for PNR and the PNR value obtained in our study ranged from 7.09 to 78.74, it indicated that a unitary change in PNR has little clinical significance. Therefore, we reduced the PNR values 10-fold in the final model. As shown in Table 4, every increase in PNR by 10 units led to a 43% increased risk of MACEs (95% CI: 1.16–1.75 on the right side of the inflection point), whereas a possible opposite trend on the left side of the inflection point existed, but with no statistical significance observed.

## 4. Discussion

Our findings indicated that PNR was positively associated with MACEs in STEMI patients who underwent successful pPCI after adjusting for confounding factors. Furthermore, the threshold effect was revealed through further analyses. To the best of our knowledge, this is the first report to provide a clear, quantified, nonlinear effect of PNR on the incidence of MACEs in post-pPCI STEMI patients.

Only one previous study has evaluated the relationship between the neutrophil-to-platelet ratio (NPR), which is the inverse of the PNR we investigated, and the short-term prognosis of STEMI after pPCI [14]. In that study, an NPR > 0.045 (which corresponds with PNR <22.2) was correlated with increased 30-day mortality in STEMI patients after pPCI [14]. Interestingly, we achieved a similar inflection point (23.1 in this study). We also observed a potentially negative trend when PNR < 23.1, although insignificant. Unfortunately, the detailed results of that study are unavailable; hence, further comparisons could not be performed. We anticipate that more studies can further verify the results of this study in future.

Atherosclerosis, chronic inflammation, and acute plaque disruption play essential pathological roles in most STEMI patients [19, 20]. The prognosis of patients with STEMI is influenced not only by the severity of acute local lesions but also by the preexisting and persistent chronic inflammatory condition of atherosclerosis [5, 16, 19]. Emerging studies have defined platelet as a type of immune and inflammatory cell [13, 15] involved in every pathological aspect of atherosclerosis [21–24]. Its level reflects the condition of preexisting inflammation in coronary artery disease (CAD). Meanwhile, neutrophil count is closely correlated with the severity of CAD [25–27]. In our study, PNR was positively associated with the long-term prognosis of STEMI after successful pPCI, which indicated that preexisting inflammatory conditions expressed by fluctuating platelet levels might play a relatively more important role than acute inflammatory reactions, which were expressed by neutrophils in STEMI. Our results, on the one hand, demonstrated the necessity of primary and secondary prevention of CAD; on the other hand, they might provide new approaches for the management of STEMI.

We recognize that this study had the following limitations: (1) This study was conducted on patients with STEMI who had undergone successful pPCI. Therefore, timely and effective revascularization by PCI might weaken the damage caused by acute injury and alleviate the impact of acute inflammation on clinical outcomes. (2) Since patients with NSTEMI were excluded from this study, the findings of this study cannot be used for these patients. (3) As data limited, no other detailed procedural data,such as incidence of no-reflow and application of thrombus aspiration, thepotential influence brought by these limitations of secondary analysis couldnot be evaluated further. Fortunately, the original study reported that no significant difference existed between the MACE and non-MACE groups, which was also insignificant in the subsequent Cox regression. Furthermore, all enrolled patients underwent successful pPCI. This would, to some extent, alleviate possible heterogeneity among participants. Future research is needed to explore the influence of the balance between chronic inflammation and acute inflammation on the prognosis of patients with STEMI after undergoing pPCI.

## 5. Conclusion

There is a threshold relationship between PNR and MACEs in patients with STEMI after successful pPCI. The incidence of MACEs was positively associated with PNR when the PNR exceeded 23.1.

## Figures and Tables

**Table 1:** Clinical characteristic of participants.

PNR	Q1 (7.09-22.83) *N* = 116	Q2 (22.90-31.46) *N* = 115	Q3 (31.49-43.15) *N* = 115	Q4 (43.41-78.74) *N* = 116	*p* value	*p* value^∗^
Age (years, mean ± sd)	62.37 ± 11.45	63.22 ± 11.13	63.17 ± 13.01	63.13 ± 12.12	0.941	0.941
Gender (male, *n* (%))	86 (74.14%)	85 (73.91%)	84 (73.04%)	99 (85.34%)	0.085	—
HBP (*n*, %)	72 (62.07%)	70 (60.87%)	59 (51.30%)	63 (54.31%)	0.286	—
DM (*n*, %)	38 (32.76%)	41 (35.65%)	30 (26.09%)	40 (34.48%)	0.409	—
Myocardial infarction history (*n*, %)	14 (12.07%)	15 (13.04%)	10 (8.70%)	16 (13.79%)	0.644	—
Anterior wall myocardial infarction (*n*, %)	60 (51.72%)	45 (39.13%)	64 (55.65%)	61 (52.59%)	0.062	—
SBP (mmHg, mean ± sd)	134.74 ± 25.37	130.06 ± 29.81	133.23 ± 26.00	129.29 ± 27.19	0.371	0.347
HR (bpm, mean ± sd)	77.58 ± 17.69	75.44 ± 16.75	78.38 ± 17.41	76.42 ± 16.74	0.582	0.571
WBC (×10^9^/L, mean ± sd)	13.45 ± 2.58	11.36 ± 3.16	9.00 ± 2.38	6.52 ± 1.96	<0.001	<0.001
Neutrophil (%, mean ± sd)	81.65 ± 9.41	75.32 ± 10.46	74.56 ± 10.36	71.33 ± 13.14	<0.001	<0.001
Hemoglobin (g/L, mean ± sd)	144.96 ± 16.95	143.28 ± 17.31	143.36 ± 17.57	143.49 ± 17.07	0.863	0.860
Platelet (×10^9^/L, mean ± sd)	192.69 ± 43.64	227.93 ± 52.12	239.54 ± 52.21	267.74 ± 49.61	<0.001	<0.001
PNR	17.95 ± 3.44	27.34 ± 2.47	36.59 ± 3.70	63.13 ± 21.88	<0.001	<0.001
BUN (mmol/L, mean ± sd)	6.80 ± 2.07	6.61 ± 1.98	6.89 ± 2.18	6.64 ± 2.08	0.703	0.680
Creatinine (mmol/L, mean ± sd)	72.38 ± 14.08	73.82 ± 15.93	76.80 ± 35.16	75.56 ± 19.68	0.473	0.839
eGFR (mL/min × 1.73 m^2^, mean ± sd)	0.43 ± 0.12	0.42 ± 0.12	0.43 ± 0.15	0.42 ± 0.13	0.935	0.940
Uric acid (mmol/L, mean ± sd)	329.83 ± 74.30	342.32 ± 74.45	340.16 ± 71.65	336.20 ± 74.79	0.591	0.556
Albumin (g/L, mean ± sd)	37.26 ± 3.94	38.53 ± 3.58	38.41 ± 3.67	37.58 ± 4.05	0.029	0.040
FBG (mmol/L, mean ± sd)	7.34 ± 2.50	7.80 ± 2.50	7.93 ± 2.61	7.59 ± 2.51	0.309	0.275
TG (mmol/L, median (quartile))	1.06 (0.61-1.56)	1.00 (0.52-1.42)	0.98 (0.59-1.54)	1.07 (0.60-1.63)	0.378	0.752
TC (mmol/L, mean ± sd)	5.78 ± 1.24	5.64 ± 1.12	5.55 ± 1.09	5.60 ± 1.09	0.477	0.344
HDL-C (mmol/L, mean ± sd)	1.20 ± 0.29	1.20 ± 0.25	1.18 ± 0.28	1.23 ± 0.28	0.646	0.496
LDL-C (mmol/L, mean ± sd)	3.19 ± 0.70	3.06 ± 0.71	2.89 ± 0.70	3.04 ± 0.77	0.018	0.012
Peak cTnI (ng/mL, median (quartile))	17.45 (6.01-29.92)	15.20 (3.97-30.00)	12.50 (3.45-26.90)	11.05 (4.62-30.40)	0.376	0.337
Peak CK-MB (U/L, median(quartile))	128.50 (63.50-215.50)	112.00 (46.50-186.00)	105.00 (37.50-182.50)	94.50 (36.75-196.00)	0.085	0.050
D-Dimer (mg/L, median (quartile))	1.10 (0.40-1.80)	0.90 (0.20-1.60)	0.90 (0.20-1.50)	0.80 (0.20-1.70)	0.915	0.764
LVEDD (mm, mean ± sd)	50.81 ± 5.85	50.07 ± 5.94	49.77 ± 6.27	51.09 ± 6.97	0.335	0.262
Apelin-12 change rate (%, median (quartile))	12.00 (6.45-20.05)	14.70 (5.35-20.60)	15.50 (6.85-21.65)	13.45 (4.72-20.15)	0.451	0.472
Apelin-12 (ng/mL, mean ± sd)	0.84 ± 0.36	0.83 ± 0.32	0.84 ± 0.35	0.80 ± 0.32	0.792	0.708
Culprit vessels (*n*, %)					0.254	—
LAD	62 (53.45%)	52 (45.22%)	59 (51.30%)	58 (50.00%)		
LCX	22 (18.97%)	23 (20.00%)	14 (12.17%)	13 (11.21%)		
RCA	32 (27.59%)	40 (34.78%)	42 (36.52%)	45 (38.79%)		
Stent number (mean ± sd)	1.42 ± 0.59	1.37 ± 0.54	1.32 ± 0.49	1.41 ± 0.56	0.504	0.655
Pathological Q wave (*n*, %)	57 (49.14%)	57 (49.57%)	53 (46.09%)	55 (47.41%)	0.949	—
Killip grade > 1 (*n*, %)	34 (29.31%)	19 (16.52%)	34 (29.57%)	25 (21.55%)	0.055	—
GENSINI	76.32 ± 30.20	69.91 ± 33.32	69.57 ± 33.53	71.82 ± 31.77	0.360	0.361
MACE (*n*, %)	33 (28.45%)	27 (23.48%)	28 (24.35%)	30 (25.86%)	0.834	—
Survival time (months, median (quartile))	25.04 (6.20-28.61)	27.72 (15.79-29.25)	23.12 (0.84-26.62)	21.38 (0.51-25.82)	0.228	0.006

Abbreviations: BUN: blood urea nitrogen; CK-MB: creatine kinase MB; cTnI: cardiac troponin I; eGFR: estimated glomerular filtration rate; FBG: fasting blood glucose; HDL-C: high-density lipoprotein cholesterol; LAD: left anterior descending branch; LCX: left circumflex coronary artery; LDL-C: low-density lipoprotein cholesterol; LVEDD: left ventricular and diastolic diameter; MACEs: major adverse cardiovascular events; MI: myocardial infarction; RCA: right coronary artery; SBP: systolic blood pressure; TC: total cholesterol; TG: triglyceride; WBC: white blood cells. ^∗^*p* value calculated by Kruskal-Wallis H test (skewed distribution).

**Table 2:** The results of univariate analysis.

	Statistics	Effect size (HR, 95% CI)	*p* value
Age	66.95 ± 12.16	1.01 (0.99, 1.02)	0.2427
Gender			
Female	33 (27.97%)	Ref	
Male	85 (72.03%)	1.55 (1.03, 2.33)	0.0353
HBP			
No	46 (38.98%)	Ref	
Yes	72 (61.02%)	0.90 (0.62, 1.31)	0.5881
DM			
No	77 (65.25%)	Ref	
Yes	41 (34.75%)	0.86 (0.59, 1.27)	0.4525
Previous MI			
No	99 (83.90%)	Ref	
Yes	19 (16.10%)	1.25 (0.76, 2.06)	0.3739
Anterior wall MI			
No	46 (38.98%)	Ref	
Yes	72 (61.02%)	1.01 (0.69, 1.46)	0.9723
SBP	132.22 ± 26.59	1.00 (0.99, 1.01)	0.8123
HR	79.48 ± 18.51	1.00 (0.99, 1.02)	0.3959
WBC	10.60 ± 3.86	0.84 (0.79, 0.89)	<0.0001
Neutrophil	76.76 ± 12.56	1.01 (0.99, 1.02)	0.3747
Hemoglobin	139.42 ± 16.66	1.00 (0.99, 1.01)	0.9739
Platelet	240.18 ± 60.08	1.00 (1.00, 1.00)	0.3973
PNR	34.90 ± 17.25	1.02 (1.01, 1.04)	<0.0001
BUN	6.78 ± 1.88	0.97 (0.88, 1.07)	0.5292
Creatinine	76.28 ± 15.64	1.02 (1.01, 1.03)	0.0031
eGFR	0.40 ± 0.12	0.21 (0.05, 0.91)	0.0374
Uric acid	333.34 ± 80.66	1.00 (1.00, 1.00)	0.0440
Albumin	37.88 ± 3.91	0.99 (0.94, 1.04)	0.7218
FBG	7.67 ± 2.68	0.96 (0.90, 1.03)	0.2573
TG	1.08 ± 0.65	0.86 (0.64, 1.14)	0.2891
TC	5.87 ± 0.99	1.16 (0.95, 1.41)	0.1450
HDL-C	1.25 ± 0.28	1.21 (0.59, 2.48)	0.6058
LDL-C	3.07 ± 0.72	0.92 (0.71, 1.21)	0.5636
Peak cTnI	18.47 ± 11.75	0.99 (0.98, 1.01)	0.4161
Peak CK-MB	133.20 ± 88.07	1.00 (1.00, 1.00)	0.9251
D-dimer	0.97 ± 0.88	0.92 (0.73, 1.16)	0.4907
LVEDD	51.98 ± 6.39	1.03 (1.00, 1.06)	0.0552
Apelin-12	0.71 ± 0.28	0.74 (0.38, 1.43)	0.3739
Apelin-12 change rate	12.31 ± 7.31	1.01 (0.99, 1.03)	0.4852
Culprit vessels			
LAD	64 (54.24%)	Ref	
LCX	18 (15.25%)	0.95 (0.56, 1.62)	0.8642
RCA	36 (30.51%)	1.18 (0.78, 1.78)	0.4428
Stent number	1.35 ± 0.55	1.06 (0.73, 1.54)	0.7415
Pathological Q wave			
No	48 (40.68%)	Ref	
Yes	70 (59.32%)	1.09 (0.75, 1.58)	0.6475
Killip classification > 1			
No	77 (65.25%)	Ref	
Yes	41 (34.75%)	1.47 (1.00, 2.17)	0.0509
GENSINI	75.98 ± 30.46	1.00 (0.99, 1.01)	0.7981

**Table 3:** Impacts of PNR on MACEs in different models.

Variable	Crude model (HR, 95% CI, *p*)	Minimally adjusted model (HR, 95% CI, *p*)	Fully adjusted model (HR, 95% CI, *p*)
PNR	1.03 (1.02, 1.05) <0.0001	1.03 (1.02, 1.04) <0.0001	1.02 (1.00, 1.04) 0.0152
PNR (quartile)			
Q1	Ref	Ref	Ref
Q2	0.79 (0.47, 1.33) 0.3743	0.65 (0.38, 1.12) 0.1225	0.64 (0.35, 1.18) 0.1500
Q3	1.91 (1.13, 3.24) 0.0156	1.95 (1.15, 3.31) 0.0129	1.69 (0.91, 3.14) 0.0971
Q4	2.70 (1.55, 4.72) 0.0005	2.44 (1.39, 4.26) 0.0018	2.04 (1.07, 3.88) 0.0307
P for trend	1.44 (1.19, 1.74) 0.0002	1.42 (1.17, 1.71) 0.0003	1.29 (1.04, 1.61) 0.0223

Crude model: we did not adjust other covariates. Minimally adjusted model: we adjusted age and sex. Fully adjusted model: we adjusted age, sex, anterior wall myocardial infarction, myocardial infarction history, apelin-12, apelin-12 change rate, LVEDD, peak cTnI, pathological Q wave, Killip grade, FBG, albumin, GENSINI and eGFR. CI confidence interval, Ref reference.

**Table 4:** The results of two-piecewise linear regression model.

Inflection point of PNR	Effect size (HR)	95% CI	*p* value
<2.31	0.57	0.29 to 1.13	0.1084
≥2.31	1.43	1.16 to 1.75	0.0006

Effect: MACEs. Cause: PNR (per 10-unit change). Adjusted: age, sex, anterior wall myocardial infarction, myocardial infarction history, apelin-12, apelin-12 change rate, LVEDD, peak cTnI, pathological Q wave, Killip grade, FBG, albumin, GENSINI, and eGFR.

## Data Availability

Data can be accessed from “DATADRYAD” database (https://datadryad.org/stash/dataset/doi:10.5061/dryad.pf56m).
